# Supplementary material for: Sustained-input switches for transcription factors and microRNAs are central building blocks of eukaryotic gene circuits
Source: Genome Biol. 2013 Aug 23;14(8):R85. doi: 10.1186/gb-2013-14-8-r85 (PMC4054853; doi:10.1186/gb-2013-14-8-r85)
Supplement: Additional file 5 — HTML Browsable Motif Output. Zipped folder containing all WaRSwap and FANMOD motif output, viewable in a web browser. [file gb-2013-14-8-r85-S5.ZIP › HTML_browsable_motif_output/WaRSwap_ath_tair10/sigs_WSWRSLDraw_TAIR10-2500.pvals.heatmaps.html/motif_id_14_010111002_tftype_ath_upstream_-3000_0.html]

```
BG_MODEL = WARSwap
MOTIF_ID = 14_010111002
TF_TYPE = ath
UPSTREAM = -3000_0


PVals
FNR = 0.2	FNR = 0.4	FNR = 0.6	FNR = 0.8
deltaG = 60	0.029	0.014	0.008	0.53
deltaG = 70	0.005	0.01	0.002	0.467
deltaG = 80	0.009	0.004	0.002	0.342

ZScores
FNR = 0.2	FNR = 0.4	FNR = 0.6	FNR = 0.8
deltaG = 60	2.882	3.238	3.717	-0.312
deltaG = 70	3.761	3.726	4.595	-0.229
deltaG = 80	3.601	3.833	4.265	-0.238

StDevs
FNR = 0.2	FNR = 0.4	FNR = 0.6	FNR = 0.8
deltaG = 60	148.901	150.527	145.367	101.448
deltaG = 70	127.182	139.527	123.106	91.627
deltaG = 80	83.434	85.238	81.441	64.021
```
